# CRISPR interference‐mediated gene regulation in *Pseudomonas putida* KT2440

**DOI:** 10.1111/1751-7915.13382

**Published:** 2019-02-22

**Authors:** Seong Keun Kim, Paul K. Yoon, Soo‐Jung Kim, Seung‐Gyun Woo, Eugene Rha, Hyewon Lee, Soo‐Jin Yeom, Haseong Kim, Dae‐Hee Lee, Seung‐Goo Lee

**Affiliations:** ^1^ Synthetic Biology and Bioengineering Research Center Korea Research Institute of Bioscience and Biotechnology (KRIBB) Daejeon 34141 Korea; ^2^ Department of Biosystems and Bioengineering KRIBB School of Biotechnology University of Science and Technology (UST) Daejeon 34113 Korea

## Abstract

Targeted gene regulation is indispensable for reprogramming a cellular network to modulate a microbial phenotype. Here, we adopted the type II CRISPR interference (CRISPRi) system for simple and efficient regulation of target genes in *Pseudomonas putida* KT2440. A single CRISPRi plasmid was generated to express a nuclease‐deficient Cas9 gene and a designed single guide RNA, under control of l‐rhamnose‐inducible P_rha_ _BAD_ and the constitutive Biobrick J23119 promoter respectively. Two target genes were selected to probe the CRISPRi‐mediated gene regulation: exogenous green fluorescent protein on the multicopy plasmid and endogenous *glpR* on the *P. putida* KT2440 chromosome, encoding GlpR, a transcriptional regulator that represses expression of the *glpFKRD* gene cluster for glycerol utilization. The CRISPRi system successfully repressed the two target genes, as evidenced by a reduction in the fluorescence intensity and the lag phase of *P. putida* KT2440 cell growth on glycerol. Furthermore, CRISPRi‐mediated repression of *glpR* improved both the cell growth and glycerol utilization, resulting in the enhanced production of mevalonate in an engineered *P. putida* KT2440 harbouring heterologous genes for the mevalonate pathway. CRISPRi is expected to become a robust tool to reprogram *P. putida* KT2440 for the development of microbial cell factories producing industrially valuable products.

## Graphical abstract

We present an l‐rhamnose‐inducible single‐plasmid CRISPRi system for achieving the simple and efficient regulation of target genes in *Pseudomonas putida* KT2440. This regulatable CRISPRi system was able to control the expression of exogenous and endogenous genes in *P*. *putida* KT2440. We further provide examples of its application for metabolic flux alteration to enhance the production of mevalonate (MVA), a key intermediate metabolite for the biosynthesis of a myriad of terpenoids.

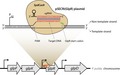

## Introduction

*Pseudomonas putida* KT2440 is a Gram‐negative soil bacterium that is classified as a generally regarded as safe (GRAS)‐certified strain (Loeschcke and Thies, 2015; Nikel and de Lorenzo, 2018). This strain has attracted substantial attention for industrial applications owing to its fast growth, native metabolic versatility and high robustness in harsh environmental conditions, which are particularly suitable traits for bio‐remediation of environmental contamination and metabolic engineering (Nikel *et al*., 2016; Nikel and de Lorenzo, 2018). In addition, genome‐scale metabolic models of *P. putida* KT2440 were developed to better understand the versatile metabolism of the strain at the systems level, which were applied to reprogram the phenotype for biotechnological implementations (Nogales *et al*., 2008; Sohn *et al*., 2010; Hintermayer and Weuster‐Botz, 2017). Indeed, well‐established molecular biological tools, including systems for gene expression and genome engineering, have been harnessed in *P. putida* KT2440 to produce biofuels and pharmaceuticals by metabolic engineering (Martínez‐García and de Lorenzo, 2011; Cook *et al*., 2018).

Although *P. putida* KT2440 has been widely used as a chassis for metabolic engineering, synthetic biology and bio‐remediation, there is no reliable and standardized method for the sequence‐specific regulation of genes of interest. Recently, clustered regularly interspaced palindromic repeats (CRISPR) and CRISPR‐associated (Cas) protein have emerged as versatile genome editing tools for diverse organisms, including bacteria (Cho *et al*., 2018), mammalian cells (Komor *et al*., 2017) and plants (Ma *et al*., 2016). Besides genome editing, the CRISPR‐Cas system was repurposed to control gene expression in a method referred to as CRISPR interference (CRISPRi) (Qi *et al*., 2013). Like CRISPR‐Cas systems, CRISPRi also requires a nuclease‐deficient Cas9 (dCas9) protein and a specifically designed single guide RNA (sgRNA) comprising a 20‐nucleotide (nt) spacer sequence complementary to the target DNA sequence. The dCas9‐sgRNA complex then binds to the strand of target DNA, which interferes with transcription initiation or elongation by blocking RNA polymerase binding (Qi *et al*., 2013). The CRISPRi system has also been widely used as an exceptionally simple and efficient tool for sequence‐specific regulation of target genes in a number of organisms, including *Escherichia coli* (Qi *et al*., 2013), *Bacillus subtilis* (Peters *et al*., 2016) and mammalian cells (Gilbert *et al*., 2013).

CRISPRi‐based gene regulation has also been reported for some *Pseudomonas* spp. Tan *et al*. (2018) developed a tunable CRISPRi system for realizing dynamic gene repression in *Pseudomonas aeruginosa* using the type II dCas9 homologue of *Streptococcus pasteurianus*. Specifically, they constructed two plasmids to express the *dCas9* gene and sgRNA from a P_lac_ and P_tet_ promoter respectively. However, the developed two‐plasmid CRISPRi system showed leaky expression of the *dCas9* gene in *P. aeruginosa*, which resulted in repression of the target gene in the absence of the inducer. Moreover, the type I CRISPR‐dCas system was adopted as a transcriptional repressor in *P. aeruginosa* PA14, which requires deletion of the *Cas3* gene from *P. aeruginosa* or expression of anti‐CRISPR proteins from a prophage (Bondy‐Denomy *et al*., 2015). More recently, a CRISPRi technique was developed using dCas9 of *Streptococcus pyogenes* (*Sp*dCas9), which demonstrated functionality using enhanced green fluorescent protein (eGFP) in *P. putida* KT2440 (Sun *et al*., 2018). However, this study lacked CRISPRi applications to metabolic engineering or synthetic biology of *P. putida* KT2440.

Here, we present an l‐rhamnose‐inducible single‐plasmid CRISPRi system for achieving the simple and efficient regulation of target genes in *P. putida* KT2440. This regulatable CRISPRi system was able to control the expression of exogenous and endogenous genes in *P. putida* KT2440. We further provide examples of its application for metabolic flux alteration to enhance the production of mevalonate (MVA), a key intermediate metabolite for the biosynthesis of a myriad of terpenoids. *Pseudomonas putida* KT2440 shows a prolonged lag phase on glycerol as the sole carbon source; thus, strategies to reduce this lag phase could help to overcome this limitation of the strain in using a cost‐effective renewable resource for the microbial production of terpenoids. Using a key regulator involved in glycerol metabolism of *P. putida* KT2440 as a target gene for the single‐plasmid CRISPRi system proved to be a robust platform for modulation of endogenous gene expression. This system can therefore accelerate the metabolic engineering of *P. putida* KT2440 for the development of microbial cell factories that can produce industrially valuable products in the future.

## Protocol

### Overview of the CRISPRi system

**Figure 1:**
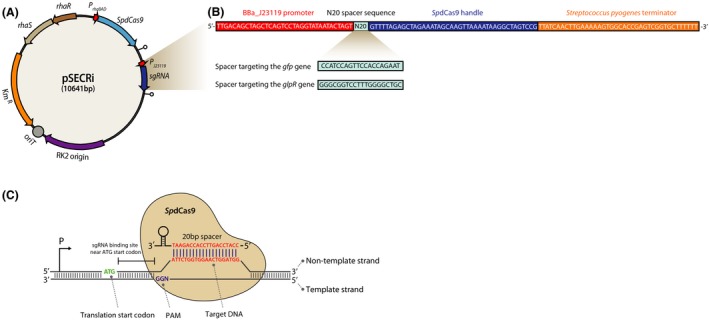
A single‐plasmid‐based CRISPRi system in *Pseudomonas putida* KT2440. (A) The CRISPRi expression plasmid, pSECRi. The CRISPRi system consists of an l‐rhamnose‐inducible *Sp*dCas9 protein and a designed sgRNA chimera encompassing a J23119 constitutive promoter in a low copy (RK2 origin) pSEVA221 plasmid. (B) The architecture of customized sgRNAs with the constitutive J23119 promoter. The sgRNA consists of base‐pairing nucleotides for specific target DNA sequence binding (N20 spacer sequence, 20 bp), a 42 bp *Sp*dCas9‐binding handle and 40 bp *Streptococcus pyogenes* terminator. A plasmid‐borne green fluorescent protein (GFP) and endogenous GlpR were chosen as target sites. (C) Schematic representation of CRISPRi targeting the gene of interest. A 20 bp sgRNA spacer sequence targeting the gene was designed according to the following criteria; (i) the 3′ end of the target region should contain a proto‐spacer adjacent motif (PAM) sequence (5′‐NGG‐3′), (ii) the sgRNA should bind to the non‐template DNA strand proximal to the ATG translational start codon of the target gene for high repression efficiency.

To develop a CRISPRi system in *P. putida* KT2440, we first created a single pSECRi plasmid that expresses catalytically inactive *Sp*dCas9 and a target‐specific sgRNA (Fig. 1A). By designing a 20 bp spacer sequence to bind the target sequence (N20 spacer sequence, Fig. 1B), the *Sp*dCas9‐sgRNA complex can be recruited to the gene of interest, leading to transcriptional repression. For expression of the *Sp*dCas9 protein and sgRNA, the low copy number pSEVA221 plasmid (Kues and Stahl, 1989) was used as the backbone plasmid for generating pSECRi, which minimized metabolic burden and allowed other antibiotic‐resistance genes and replication origins to be swapped for use in applications with other bacterial hosts (Silva‐Rocha *et al*., 2013). To enable tunable control of gene repression in *P. putida* KT2440, the *SpdCas9* gene was first placed under control of the l‐rhamnose‐inducible promoter (P_rhaBAD_) including RhaR and RhaS regulators, whereas sgRNA expression was driven by a constitutive BBa_J23119 promoter (http://parts.igem.org/Part:BBa_J23119, hereafter named as J23119) on a pSEVA221‐derived plasmid. P_rhaBAD_ is a tightly regulated promoter among various inducible promoters including isopropyl β‐D‐1‐thiogalactopyranoside (IPTG)‐inducible (P_lacUV5_, P_T7_) and XylS/P_m_ promoters that have been widely used in *P. putida* KT2440 for the expression of heterologous genes in metabolic engineering efforts (Calero *et al*., 2016). In *E. coli*, the RhaS and RhaR activators of the P_rhaBAD_ promoter in the CRISPRi plasmid can be removed to reduce the plasmid size without changing functionality (Wegerer *et al*., 2008). However, RhaS and RhaR activators are essential to the functionality of the P_rhaBAD_ promoter in *P. putida* KT2440 (Jeske and Altenbuchner, 2010). Therefore, we chose the l‐rhamnose‐inducible and J23119 promoters as an orthogonal control for transcription of the *SpdCas9* gene and sgRNA, respectively, in the present CRISPRi system.

### General workflow for CRISPRi

To implement CRISPRi for repression of target genes, sgRNA design, cloning and expression are carried out. The general CRISPRi steps for *P. putida* KT2440 using the pSECRi harbouring the *Sp*dCas9 are summarized as follows: (i) choose the target DNA sequence to be repressed using available *Pseudomonas* genome databases (http://www.pseudomonas.com) or National Center for Biotechnology Information (NCBI, https://www.ncbi.nlm.nih.gov); (ii) determine the DNA loci to be targeted. For efficient repression, the non‐template DNA strand of the 5ʹ region, the 5ʹ UTR (untranslated region), or both DNA strand of the promoter region (‐35 and ‐10 boxes) can be chosen; (iii) select the base‐pairing sequence of the sgRNA (C20, the complementary sequence of N20 in Fig. 1B). To bind the non‐template strand of the target DNA sequence, search the transcribed sequence for 5ʹ‐CCN‐C20‐3ʹ. CCN (5ʹ‐NGG‐3ʹ on the template strand) is the proto‐spacer adjacent motif (PAM) sequence of *Sp*dCas9 in the pSECRi plasmid (Fig. 1C). The reverse complementary sequence of C20 should be used as the spacer sequence of the sgRNA; (iv) If necessary, check for off‐targets using artemis Software (https://www.sanger.ac.uk/science/tools/artemis). Using the 12 bp seed sequence and PAM sequence, perform a ‘base pattern’ search against the whole *Pseudomonas* genome. Use only sgRNA sequences without any predicted off‐targets (see *sgRNA design for CRISPRi* section); and (v) clone the sgRNA into the pSECRi plasmid under the synthetic J23119 constitutive promoter (see *Construction of the CRISPRi plasmid* section).

### sgRNA design for CRISPRi

For the application of CRISPRi, the first step is designing the spacer sequence of sgRNA to regulate the gene of interest. If information regarding the promoter location is available, it is possible to design the sgRNA‐binding site to prevent transcription initiation, but in general, it is easier to select the sgRNA‐binding site in the coding sequence (CDS) of the target gene to block transcriptional elongation. For *Sp*dCas9, the 3′ end of the target region should contain a PAM sequence (5′‐NGG‐3′) and a 20 bp spacer is adequate for efficient repression of the target gene expression (Fig. 1C) (Qi *et al*., 2013). For CRISPRi, a critical point is that the sgRNA should bind to the non‐template DNA strand proximal to the ATG translational start codon of the target gene for high repression efficiency (Qi *et al*., 2013; Kim *et al*., 2017). Although off‐target effects are less concerning for CRISPRi, a genome‐wide search for matching sequences with the PAM‐proximal 12 bp ‘seed’ region including 5′‐NGG‐3′ PAM might be helpful for improving the specific repression of target genes (Bikard *et al*., 2013). For this purpose, we used artemis Software to analyse potential off‐target sites against the *P. putida* KT2440 genome sequence (NCBI number: NC_002947) by searching genes that contain the 15 bp sequence (i.e. 12 bp seed sequence of the sgRNA and 3 bp of the 5′‐NGG‐3′ PAM sequence).

### Bacterial strains, media and culture conditions

*Escherichia coli* DH5α was used for cloning experiments and gene expression analysis at the single‐cell level of the P_rhaBA*D*_ promoter. *Pseudomonas putida* KT2440 was used for all CRISPRi experiments. Bacteria were cultured in lysogeny broth (LB, 10 g l^−1^ tryptone, 5 g l^−1^ yeast extract and 10 g l^−1^ NaCl) at 30°C. For *glpR* gene repression, *P. putida* KT2440 was grown on M9 minimal medium (6.78 g l^−1^ Na_2_HPO_4_, 3 g l^−1^ KH_2_PO_4_, 0.5 g l^−1^ NaCl, 1 g l^−1^ NH_4_Cl, 0.241 g l^−1^ MgSO_4_ and 2.5 ml l^−1^ A9 solution) (Abril *et al*., 1989) with 4 g l^−1^ glycerol as the carbon source and appropriate antibiotics at 30°C. For CRISPRi‐based gene repression, 1 mM l‐rhamnose was added to the culture medium. IPTG was added to the culture medium at 0.1 mM unless otherwise noted. All chemicals were purchased from Sigma‐Aldrich (St Louis, MO, USA).

### Construction of reporter and MVA plasmids

The plasmids and primers used in this study are listed in Table 1 and Table 2 respectively. All restriction enzymes, T4 DNA ligase, T4 polynucleotide kinase and Gibson Assembly Master Mix were purchased from New England Biolabs (Ipswich, MA, USA). KOD‐Plus‐Neo polymerase (Toyobo, Osaka, Japan) was used for high‐fidelity polymerase chain reaction (PCR), and colony PCR was performed using AccuPower^®^ PCR PreMix (Bioneer, Daejeon, Korea). The plasmid miniprep and DNA purification kits were obtained from Promega (Madison, WI, USA). Oligonucleotides were synthesized by Macrogen (Seoul, Korea). All experiments were conducted according to the manufacturer's instructions.

**Table 1:** Plasmids used in this study

Plasmid	Description	Reference
pSEVA221	RK2 ori, Km^*R*^	Silva‐Rocha *et al*. (2013)
pSEVA231	pBBR1 ori, Km^*R*^	Silva‐Rocha *et al*. (2013)
pSEVA631	pBBR1 ori, Gm^*R*^	Silva‐Rocha *et al*. (2013)
pK7‐sfGFP	sfGFP expressing plasmid in pK7	Lee *et al*. (2016)
pSNA‐MrBBS‐IspA	*lacI* ^*q*^‐P_*trc*_‐*MrBBS*‐*ispA*‐P_*lac*_‐*mvaK1‐mvaK2‐mvaD‐idi‐mvaE‐mvaS* in pTrc99a	Han *et al*. (2016)
pSECRi	*rhaRS*‐P_*rhaBAD*_‐*cas9*(D10A, H840A) and constitutive sgRNA expression cassette in pSEVA221	Kim *et al*. (2016)
pSR‐GFP	*rhaRS*‐P_*rhaBAD*_‐*gfp* in pSEVA221, RK2 ori, Km^*R*^	This study
pT‐GFP	*lacI* ^*q*^‐P_*trc*_‐*gfp* in pTrc99a	This study
pST‐GFP	*lacI* ^*q*^‐P_*trc*_‐*gfp* in pSEVA631, pBBR1 ori, Gm^*R*^	This study
pST‐BISA	*lacI* ^*q*^‐ P_*trc*_‐*MrBBS*‐*ispA*‐P_*lac*_‐*mvaK1‐mvaK2‐mvaD‐idi‐mvaE‐mvaS* in pSEVA231, pBBR1 ori, Km^*R*^	This study
pST‐MVA	*lacI* ^*q*^‐P_*trc*_‐*mvaE‐mvaS* in pSEVA231, pBBR1 ori, Km^*R*^	This study
pSECRi(GFP)	pSECRi plasmid targeting a *gfp* gene, RK2 ori, Km^*R*^	This study
pSECRi(GlpR)	pSECRi plasmid targeting a *glpR* gene, RK2 ori, Km^*R*^	This study
pSECRi(GlpR)‐Gen	pSECRi plasmid targeting a *glpR* gene, RK2 ori, Gm^*R*^	This study

**Table 2:** Primers used in this study

Primer	Oligonucleotide sequence (5ʹ to 3ʹ)	Plasmid construction
GFP‐VF	ctttgctcatatggtgatcctgctgaattt	pSR‐GFP
GFP‐VR	cgaaaaataagcggccgcctcgaggaagct	
GFP‐IF	aggcggccgcttatttttcgaactgcggat	
GFP‐IR	ggatcaccatatgagcaaaggtgaagaact	
TG‐VF	cgaaaaataatctagagtcgacctgcaggc	pST‐GFP
TG‐VR	ctttgctcatggtttaacctcctgtgtgaa	
TG‐IF	aggttaaaccatgagcaaaggtgaagaact	
TG‐IR	cgactctagattatttttcgaactgcggat	
STG‐IF	gcctaggccgcggccgcgcggaaggcgaagcggcatgcat	
STG‐IR	ccagtcacgacgcggccgcaaagagtttgtagaaacgcaa	
STB‐VF	ttgcgtttctacaaactcttgtcgtgactgggaaaaccct	pST‐MVA
STB‐VR	cgtaaatgcatgccgcttcgtcctgtgtgaaattgttatc	
STB‐IF	gataacaatttcacacaggacgaagcggcatgcatttacg	
STB‐IR	agggttttcccagtcacgacaagagtttgtagaaacgcaa	
MVA‐F	cctcctgtgtgaaattgttatccgctcacaattcc	
MVA‐R	ttaaaccatgaaaacagtagttattattgatgc	
CRI(GFP)‐F	ccatccagttccaccagaatgttttagagctagaaatagc	pSECRi(GFP)
CRI‐R	actagtattatacctaggac	
CRI(GlpR)‐F	gggcggtcctttggggctgcgttttagagctagaaatagc	pSECRi(GlpR)
CRIout‐F	acgcattgatttgagtcagc	Validation primers for colony PCR
CRIout‐R	acggcgctattcagatcct	
Seq‐R	ttttatcagaccgcttctgc	Validation primer for Sanger sequencing

#### pSR‐GFP plasmid

To construct the l‐rhamnose‐inducible GFP expression plasmid, the *sfgfp* gene was amplified from the pK7‐sfGFP plasmid using the GFP‐IF and GFP‐IR primers (Lee *et al*., 2016). The vector backbone was amplified from the pSECRi plasmid using the GFP‐VF and GFP‐VR primers (Kim *et al*., 2016; Lee *et al*., 2016). The two fragments were assembled by the Gibson assembly method, resulting in a pSR‐GFP plasmid.

#### pST‐GFP plasmid

To generate the IPTG‐inducible GFP expression plasmid, pST‐GFP, we first created a pT‐GFP plasmid as follows. The vector backbone was amplified from the pSNA‐MrBBS‐IspA plasmid using the primers TG‐VF and TG‐VR (Han *et al*., 2016; Kim *et al*., 2016), and the *sfgfp* gene was amplified from the pK7‐sfGFP plasmid using the primers TG‐IF and TG‐IR (Lee *et al*., 2016). The two fragments were assembled by the Gibson assembly method, resulting in the pT‐GFP plasmid. Using this plasmid as a template, the *gfp* cassette, including the *lacI* ^*q*^, P_trc_ promoter, *gfp* gene and *rrnB* terminator, was amplified using the primers STG‐IF and STG‐IR. The amplified fragment was assembled into the pSEVA631 plasmid prepared by digestion with EcoRI and HindIII using the Gibson assembly method, which resulted in the pST‐GFP plasmid.

#### pST‐MVA plasmid

To construct the MVA production plasmid, all MVA pathway genes (*mvaK1‐mvaD‐mvaK2‐idi‐mvaE‐mvaS*) were amplified from the pSNA‐MrBBS‐IspA plasmid using the primers STB‐IF and STB‐IR (Kim *et al*., 2016). The vector backbone was amplified from the pSEVA231 plasmid using the primers STB‐VF and STB‐VR. Two fragments were assembled by the Gibson assembly method, resulting in the pST‐BISA plasmid. To remove unnecessary genes (*mvaK1‐mvaD‐mvaK2‐idi*) from the pST‐BISA plasmid, we amplified the DNA fragment using the pST‐BISA plasmid and primers (MVA‐FYI and MVA‐R). The amplified linear DNA was gel‐purified, 5′‐phosphorylated with T4 polynucleotide kinase, and ligated with T4 DNA ligase, which yielded the pST‐MVA plasmid.

### Construction of the CRISPRi plasmid

To determine the capability of the established CRISPRi system for the regulation of heterologous genes in *P. putida* KT2440, we first selected the *gfp* gene, which is controlled by the IPTG‐inducible P_trc_ promoter from the multicopy plasmid. Next, we designed a 20‐nt sgRNA spacer sequence (5′‐CCATCCAGTTCCACCAGAAT‐3′) targeting the non‐template strand of the *gfp* gene and a 5′‐CGG‐3′ PAM sequence that was located only 33 bp away from the start codon of the gene (Fig. 3A). Then, we constructed a CRISPRi plasmid targeting the *gfp* gene of the pST‐GFP plasmid with Primer 1 (5′‐CCATCCAGTTCCACCAGAATGTTTTAGAGCTAGAAATAGC‐3′) and Primer 2 (5′‐ACTAGTATTATACCTAGGAC‐3′) synthesized from a commercial vendor. Using these primers, inverse PCR from the pSECRi plasmid template was performed using high‐fidelity DNA polymerase (Larson *et al*., 2013).

To explain the construction process of the pSECRi(GFP) plasmid in detail, we used the primers CRI(GFP)‐F and CRI‐R (Table 2) for amplification of the whole pSECRi plasmid (Lee *et al*., 2016) with high‐fidelity KOD‐Plus‐Neo polymerase under the following thermal cycling conditions: 94°C for 2 min; 35 cycles of 98°C for 10 s, 55°C for 30 s and 68°C for 10 min; and 68°C for 5 min. After PCR amplification, the amplified DNA fragment of 10.6 kb was gel‐purified using the Wizard^®^ SV Gel and PCR Clean‐Up System and the eluate was treated with DpnI at 37°C for 1 h to remove any trace of the template plasmid. The reaction mixture was further treated with T4 polynucleotide kinase and T4 DNA ligase to phosphorylate and ligate the PCR product respectively. The ligated plasmid was transformed into highly competent *E. coli* DH5α cells, and transformants were selected on LB plates containing 25 μg ml^−1^ kanamycin. The colonies were used for PCR analysis using the primers CRIout‐F and CRIout‐R with AccuPower^®^ PCR PreMix to identify positive colonies showing the 435 bp amplicon. Plasmids of the positive colonies were then prepared with the Wizard^®^ Plus SV Minipreps DNA Purification System, and Sanger sequencing was performed using the Seq‐R primer to confirm the sgRNA sequence. The pSECRi(GlpR) plasmid was constructed in the same manner using the primers CRI(GlpR)‐F and CRI‐R.

### Electroporation of plasmids into *Pseudomonas putida* KT2440

Preparation of electro‐competent cells and transformation of plasmids into *P. putida* KT2440 was performed according to a previous method with some modification (Luo *et al*., 2016). In order to prepare competent cells, a single colony of *P. putida* KT2440 was inoculated into 3 ml of LB media and grown overnight at 30°C and 200 rpm. A total of 1 ml of cultured cells was transferred to fresh 50 ml of LB media and cultured to OD600 ~0.6. The cells were harvested by centrifugation at 4°C and 5000 × g for 10 min. After washing the cells three times with ice‐cold 3 mM 4‐(2‐hydroxyethyl)‐1‐piperazineethanesulphonic acid (HEPES), they were resuspended in 250 μL of 3 mM HEPES. Electroporation was carried out in a 2 mm cuvette after adding plasmids to a 50 μL aliquot of the electro‐competent cells using the Bio‐Rad Gene Pulser Xcell™ (Bio‐Rad, Hercules, CA, USA) with settings at 2.5 kV and 200 Ω. The transformants were cultured in LB media at 30°C and 200 rpm for 2 h and then spread on LB plates with appropriate antibiotics.

### FACS‐based assay

The cultured cells were diluted with phosphate‐buffered saline (PBS) to approximately 5 × 10^6^ cells ml^−1^. Then, single‐cell fluorescence was measured using a FACScalibur™ flow cytometer (BD Biosciences, San Jose, CA, USA) equipped with a blue laser (excitation: 488 nm) and FL1 (emission: 530/30 nm) photomultiplier tube detector. To exclude debris and dead cells, correct cell populations were acquired using an FSC‐A/SSC‐A gate with 10,000 gated events recorded. bd cellquest™ pro software (BD Biosciences, San Jose, CA, USA) was used for acquisition flow cytometry, and the acquired data were analysed using the flowjo software package (FlowJo, Ashland, OR, USA).

## Examples

### Evaluation of P_rhaBAD_ promoter function

**Figure 2:**
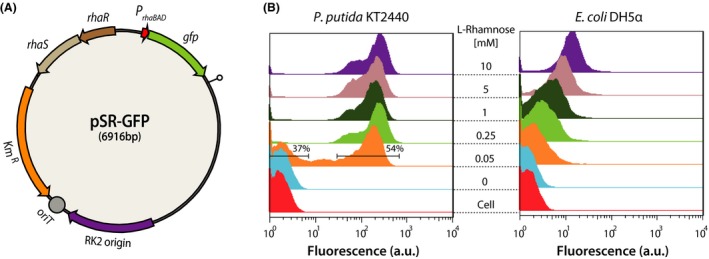
Functionality and induction phenotype of l‐rhamnose inducible promoter (P_rha_ _BAD_) in *Pseudomonas putida* KT2440. (A) A reporter plasmid, pSR‐GFP encoding the green fluorescent protein (GFP) under the control of P_rha_ _BAD_ promoter. The backbone plasmid is the same plasmid with the pSECRi plasmid. (B) Induction phenotype of P_rha_ _BAD_ promoter in *P. putida* KT2440 or *E. coli* DH5α harbouring the pSR‐GFP plasmid. Transformant cells were grown on the LB medium supplemented with various concentrations of l‐rhamnose ranging from 0–10 mM. Single‐cell fluorescence was measured by flow cytometry after growth for 12 h at 30°C. ‘Cell’ indicates *P. putida* KT2440 or *Escherichia coli* DH5α harbouring no plasmid.

We first examined the functionality of the l‐rhamnose‐inducible promoter P_rhaBAD_ that drives *Sp*dCas9 expression for the controlled regulation of the target gene. For this purpose, we replaced the *SpdCas9* gene on the pSECRi plasmid with *gfp*, which created the reporter plasmid pSR‐GFP to analyse the response of the P_rhaBAD_ promoter at the single‐cell level (Fig. 2A). The pSR‐GFP plasmid was introduced into *P. putida* KT2440 or *E. coli* DH5α, and the transformants were grown on LB medium supplemented with various concentrations of l‐rhamnose ranging from 0–10 mM. Single‐cell fluorescence was measured by flow cytometry after growth for 12 h at 30°C. As expected, the P_rhaBAD_ promoter showed tight regulation, with no leaky expression of GFP observed in the absence of l‐rhamnose in both *P. putida* KT2440 and *E. coli* DH5α (Fig. 2B). However, the *P. putida* KT2440 cells were mainly divided into two populations: non‐fluorescent (37%) or fluorescent (54%) cells in the presence of 0.05 mM l‐rhamnose (Fig. 2B). In addition, the majority of the *P. putida* KT2440 cells showed maximal green fluorescence in the presence of more than 0.25 mM l‐rhamnose, whereas the GFP fluorescence of the *E. coli* DH5α cells increased gradually and uniformly with increasing concentrations of l‐rhamnose (Fig. 2B). Based on these results, we concluded that the P_rhaBAD_ promoter showed an all‐or‐none induction phenomenon in *P. putida* KT2440. In *E. coli*, it was reported that the inability to degrade l‐rhamnose leads to the disappearance of the dose‐dependent response for P_rhaBAD_ promoter‐controlled GFP expression (Hjelm *et al*., 2017). Similarly, the response of the P_rhaBAD_ promoter was not homogenous on intermediate l‐rhamnose concentrations in *P. putida* KT2440 because l‐rhamnose might not be consumed after being imported by non‐specific sugar transporters (Jeske and Altenbuchner, 2010; Calero *et al*., 2016). Thus, the P_rhaBAD_ promoter is considered to be optimal for the on/off regulation of target genes, but not suitable for homogenous and tunable gene repression by adjusting the amount of *Sp*dCas9 through l‐rhamnose concentrations, as observed for *E. coli* (Lee *et al*., 2016). However, design of the sgRNA‐binding target region far from the ATG start codon or mismatches in the 5′ region of the spacer sequence can be employed to achieve CRISPRi‐based controllable repression (Bikard *et al*., 2013; Qi *et al*., 2013).

### CRISPRi‐mediated repression of a heterologous gene on a multicopy plasmid

**Figure 3:**
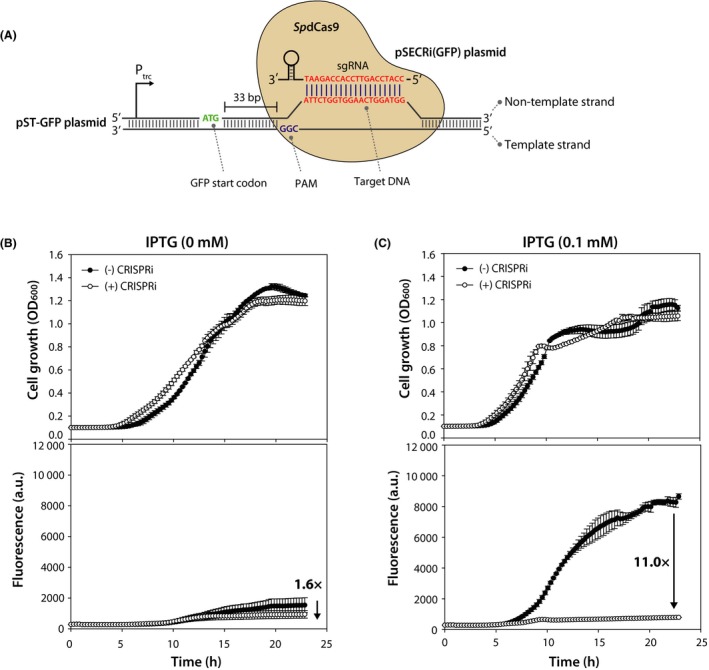
The CRISPRi‐mediated repression of a heterologous gene in *Pseudomonas putida* KT2440. (A) Schematic representation of CRISPRi targeting the green fluorescent protein (GFP) of pST‐GFP plasmid. The pST‐GFP plasmid expresses GFP under the control of IPTG‐inducible P_trc_ promoter. A 20 bp sgRNA spacer sequence targeting the *gfp* gene of pST‐GFP was designed to bind the non‐template DNA strand 33 bp away from the ATG translational start codon of the *gfp* gene. (B, C) CRISPRi‐mediated repression of plasmid‐borne *gfp* gene in *P. putida* KT2440. The pST‐GFP and pSECRi(GFP) plasmids were co‐transformed into *P. putida* KT2440, and the transformants were grown on LB medium containing 1 mM l‐rhamnose in the presence (0.1 mM) or absence of IPTG. Cell growth and GFP fluorescence were monitored simultaneously using an Infinite 200 PRO reader for 23 h at 30°C. Each graph represents the mean value of the corresponding optical density at 600 nm (OD_600_) or green fluorescence ± standard deviation of duplicate measurements from at least three independent experiments.

To determine the capability of the established CRISPRi system for regulation of heterologous genes encoded by a multicopy plasmid in *P. putida* KT2440, we constructed the GFP reporter plasmid (pST‐GFP) that was derived from the pBBR1‐based pSEVA plasmid. We chose the pBBR1 replicon because it is a broad‐host‐range plasmid and has been widely used for the production of various biochemicals in *P. putida* (Wang *et al*., 2011; Nikel and de Lorenzo, 2014; Kuepper *et al*., 2015; Yu *et al*., 2016). Then, the pST‐GFP and pSECRi(GFP) plasmids (Fig. 3A) were co‐transformed into *P. putida* KT2440, and the transformants were selected on LB plates containing both 50 μg ml^−1^ kanamycin and 20 μg ml^−1^ gentamicin at 30°C. Three single colonies were then individually inoculated into 3 ml of LB medium containing 50 μg ml^−1^ kanamycin, 20 μg ml^−1^ gentamicin and 1 mM l‐rhamnose (for *Sp*dCas9 induction), and incubated for 18 h at 30°C. Finally, 2 μL of the culture broth was inoculated into 200 μL of LB medium containing 50 μg ml^−1^ kanamycin, 20 μg ml^−1^ gentamicin, 1 mM l‐rhamnose and 0.1 mM IPTG (for GFP induction). Cell growth and GFP fluorescence were monitored simultaneously using an Infinite 200 PRO reader for 23 h at 30°C. In the absence of IPTG, GFP fluorescence slightly increased after 10 h of growth due to the leaky expression of GFP by the P_trc_ promoter. This GFP fluorescence was decreased by up to 1.6‐fold between the l‐rhamnose‐induced and uninduced CRISPRi (Fig. 3B). In the presence of 0.1 mM IPTG, the GFP fluorescence decreased by 11‐fold, nearly reaching the basal level of GFP expression (Fig. 3C).

### CRISPRi‐mediated repression of an endogenous gene

**Figure 4:**
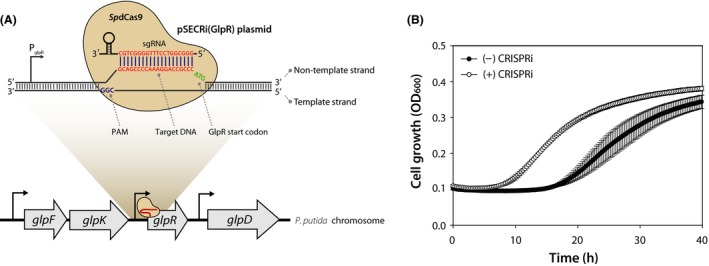
The CRISPRi‐mediated repression of an endogenous gene in *Pseudomonas putida* KT2440. (A) Schematic representation of CRISPRi targeting the endogenous GlpR regulator in *P. putida* KT2440. The endogenous GlpR regulator represses *glpFKRD* gene cluster involved in glycerol catabolism of *P. putida* KT2440. (B) CRISPRi‐mediated repression of endogenous *glpR* gene in *P. putida* KT2440. The pSECRi(GlpR) plasmid was transformed into *P. putida* KT2440, and the transformants were cultured on M9 minimal medium containing 4 g l^−1^ glycerol in the presence 1 mM l‐rhamnose. Cell growth was monitored by an Infinite 200 PRO microplate reader for 40 h at 30°C. As a control ((‐) CRISPRi), *P. putida* KT2440 harbouring the pSEVA221 plasmid was used. Each graph represents the mean value of the corresponding optical density at 600 nm (OD_600_) ± standard deviation of duplicate measurements from at least three independent experiments.

The GlpR is a transcriptional regulator that represses the expression of glycerol kinase (GlpK) and glycerol 3‐phosphate dehydrogenase (GlpD) that are responsible for the utilization of glycerol as a carbon source in *P. putida* KT2440 (Nikel *et al*., 2015). For this reason, *P. putida* KT2440 shows a prolonged lag phase on glycerol as the sole carbon source unless the *glpR* gene is deleted. To rewire the regulation of glycerol metabolism by the GlpR regulator in *P. putida* KT2440, a 20‐nt spacer sequence targeting *glpR* (5′‐GGGCGGTCCTTTGGGGCTGC‐3′) was designed and the pSECRi(GlpR) plasmid was constructed as described above using Primer 3 (5′‐GGGCGGTCCTTTGGGGCTGCGTTTTAGAGCTAGAAATAGC‐3′) and Primer 4 (5′‐ACTAGTATTATACCTAGGAC‐3′) (Fig. 4A). The constructed pSECRi(GlpR) plasmid was transformed into *P. putida* KT2440, and the transformants were selected on LB plates containing 50 μg ml^−1^ kanamycin at 30°C. Three single colonies were separately inoculated into 3 ml of LB medium containing 50 μg ml^−1^ kanamycin and 1 mM l‐rhamnose, and incubated for 18 h at 30°C. The cultured cells were washed twice with M9 minimal medium without carbon source by centrifugation and resuspended. Two microlitres of the resuspended cells were inoculated into 200 μL of M9 minimal medium containing 4 g l^−1^ glycerol, 50 μg ml^−1^ kanamycin and 1 mM l‐rhamnose, and cell growth was monitored by an Infinite 200 PRO microplate reader for 40 h at 30°C. Similar to previous reports (Escapa *et al*., 2012; Nikel *et al*., 2015), a prolonged lag phase (19 h) was observed in *P. putida* KT2440 harbouring the pSEVA221 plasmid (uninduced CRISPRi) on glycerol‐M9 minimal medium. However, the lag phase was significantly reduced from 19 h to 9 h and cell growth was accelerated when the *glpR* gene was repressed by CRISPRi (induced CRISPRi, Fig. 4B).

### Enhanced production of MVA under CRISPRi repression of glpR

**Figure 5:**
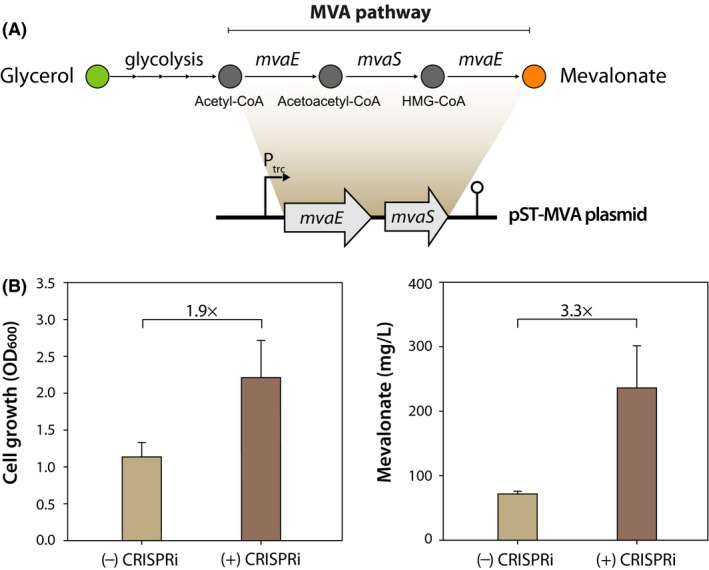
Application of the CRISPRi system for enhancing MVA production in *Pseudomonas putida* KT2440. (A) Schematic representation of the MVA production pathway and plasmid (pST‐MVA). The engineered MVA pathway encoded by pST‐MVA plasmid consists of two enzymes: MvaE, a dual function enzyme, acetoacetyl‐CoA thiolase and 3‐hydroxy‐3‐methylglutaryl‐CoA reductase of *Enterococcus faecalis*; MvaS, 3‐hydroxy‐3‐methylglutaryl‐CoA synthase of *E. faecalis*. MVA is produced by the heterologous MVA pathway from glycerol. (B) Enhanced cell growth and MVA production in *P. putida* KT2440. Both pST‐MVA and pSECRi(GlpR)‐Gen plasmids were co‐transformed into *P. putida* KT2440, and the transformants were cultured in M9 minimal medium containing 4 g l^−1^ glycerol in a 250‐ml baffled Erlenmeyer flask for 72 h at 30°C. Cell growth and mevalonate concentration were determined by spectrophotometer and HPLC respectively. As a control ((‐) CRISPRi), *P. putida* KT2440 harbouring the pSEVA221 plasmid was used. Each bar represents the mean value of the corresponding OD_600_ or MVA concentration ± standard deviation of duplicate measurements from at least three independent experiments.

Inspired by the success of *glpR* repression with our system, we next attempted to produce MVA, a precursor for sustainable biopolymers (Xiong *et al*., 2014) and terpenoids (Liao *et al*., 2016), from glycerol in *P. putida* KT2440. To this end, we constructed an MVA production plasmid (pST‐MVA), which encodes *mvaE* and *mvaS* to convert acetyl‐coA into MVA (Fig. 5A). We also generated the pSECRi(GlpR)‐Gen plasmid containing a gentamicin‐resistance gene instead of a kanamycin‐resistance gene. Both the pST‐MVA and pSECRi(GlpR)‐Gen plasmids were co‐transformed into *P. putida* KT2440, and the transformants were selected on LB plates containing 50 μg ml^−1^ kanamycin and 20 μg ml^−1^ gentamicin at 30°C. Three single colonies were separately inoculated into 3 ml of LB medium containing 50 μg ml^−1^ kanamycin, 20 μg ml^−1^ gentamicin and 1 mM l‐rhamnose, and the inoculated cells were cultivated for 18 h at 30°C. The cultured cells were washed twice in M9 minimal medium without carbon source, and 250 μL of the resuspended cells were inoculated into 25 ml M9 minimal medium containing 4 g l^−1^ glycerol, 50 μg ml^−1^ kanamycin, 20 μg ml^−1^ gentamicin and 1 mM l‐rhamnose in a 250‐ml baffled Erlenmeyer flask and cultivated for 72 h at 30°C. The culture broth was centrifuged at 3,000 rpm for 20 min at 4°C, and the supernatant was filtered through a 0.45‐μm filter. The filtrate was then used for quantifying the glycerol and MVA concentrations by high‐performance liquid chromatography (HPLC; Shimadzu, Kyoto, Japan) equipped with a refractive index detector at 454 nm using an Aminex HPX‐87H column (1300 mm × 7.8 mm, Bio‐Rad, Hercules, CA, USA). The mobile phase was sulphuric acid (0.4 mM) at a flow rate of 0.3 ml min^−1^ at 50°C. Cell growth was determined by spectrophotometry according to the optical density at 600 nm (OD_600_). Control *P. putida* KT2440 harbouring the pSEVA221 plasmid reached an OD_600_ value of 1.14, and the MVA titre was 72 mg l^−1^ on glycerol‐M9 minimal medium (Fig. 5B). However, CRISPRi‐mediated GlpR repression enhanced the cell growth by 1.9‐fold (OD_600_, 2.22) and the MVA production by 3.3‐fold (237 mg l^−1^). In addition, glycerol consumption increased from 0.96 g l^−1^ to 3.14 g l^−1^.

## Discussion

In *E. coli*, the P_rhaBAD_ promoter is capable of homogenous and l‐rhamnose‐dependent control of the transcription of heterologous genes and shows undetectable background expression in the absence of l‐rhamnose (Giacalone *et al*., 2006; Wegerer *et al*., 2008). Although the P_rhaBAD_ promoter was previously used for gene expression in *P. putida* KT2440 (Jeske and Altenbuchner, 2010), there was no report on whether or not the P_rhaBAD_ promoter is subject to a dose‐dependent homogenous expression or all‐or‐none induction phenotype under various concentrations of l‐rhamnose in *P. putida* KT2440. We found an all‐or‐none induction mode of the promoter in *P. putida* KT2440 in contrast to its effects in *E. coli*, indicating that it is not suitable for the tunable regulation of a target gene by adjusting l‐rhamnose concentrations.

In a previous report, basal expression of dCas9 due to leakiness from the inducible promoters (P_tet_, P_lac_, P_ara_) caused up to 50% repression in *Pseudomonas* spp. in the absence of inducers, which limited the basal repression of target genes (Tan *et al*., 2018). In this study, we employed the l‐rhamnose‐inducible promoter to control *Sp*dCas9 expression, which showed no leaky expression of *SpdCas9* gene (Fig. 2B). Therefore, our single‐plasmid CRISPRi system may be more effective and controllable to repress target genes without CRISPRi basal repression (Fig. 3C).

Glycerol has been used as a cost‐effective renewable resource for the production of biofuels and biochemicals, including terpenoids, because it is produced as a major by‐product of the biodiesel industry. However, the application of *P. putida* KT2440 for glycerol utilization has been limited due to the endogenous GlpR regulator that represses metabolic enzymes involved in glycerol catabolism (Nikel *et al*., 2015). Therefore, we chose the *glpR* regulator gene to be repressed by our newly developed CRISPRi system. In a previous report, *P. putida* KT2440 grown on glycerol showed a bistable growth phenotype (non‐growing and growing population), which resulted in an unexpectedly long lag phase on glycerol. GlpR knockout eliminated this bistable growth phenotype, leading to unimodal behaviour (i.e. single growing population) and reduced the lag time significantly (Nikel *et al*., 2015). In the current study, CRISPRi‐mediated *glpR* repression also reduced the lag time on glycerol remarkably, indicating that repression of an endogenous regulator gene by CRISPRi reduced phenotypic cell‐to‐cell variations, allowing *P. putida* KT2440 to better utilize glycerol as a carbon source. Compared with the conventional methods for gene knockout, the CRISPRi system has advantages as follows: (i) it is simple and easy to repress the *glpR* gene because it requires only coexpression of a *Sp*dCas9 protein and an sgRNA; (ii) it is able to repress multiple genes simultaneously including the *glpR* gene, even genes that are essential for cell growth; (iii) knockdown effects of the *glpR* gene in various *P. putida* strains can be simultaneously examined by simply introducing the single CRISPRi plasmid; and (iv) a metabolic flux towards glycerol metabolism is temporally controlled by CRISPRi‐based *glpR* repression. Thus, this system could overcome the hurdle of using *P. putida* KT2440 as microbial cell factories to produce valuable products from glycerol.

Taken together, our single‐plasmid‐based CRISPRi system developed for *P. putida* KT2440 demonstrates simplicity and efficiency for regulation of exogenous and endogenous genes. Using this system, enhanced MVA production was achieved by rewiring glycerol metabolism through CRISPRi‐mediated repression of the *P. putida* KT2440 *glpR* gene. Therefore, the CRISPRi system is a robust tool for expanding the metabolic engineering capabilities of *P. putida* KT2440, which can lead to the development of microbial cell factories.

## Conflict of interest

The authors do not have any conflict of interest.
